# Analysis of an Observational Versus Surgical Approach for Pediatric Post‐Tonsillectomy Hemorrhage

**DOI:** 10.1002/lary.70140

**Published:** 2025-09-16

**Authors:** Joshua Verhagen, Elle Nuttall, Samuel Floren, Daniel Traverzo, Tony Kille

**Affiliations:** ^1^ University of Wisconsin School of Medicine and Public Health. Madison Wisconsin USA; ^2^ Department of Otolaryngology ‐ Head and Neck Surgery University of Wisconsin–Madison Madison Wisconsin USA

**Keywords:** clinical protocol, pediatric patients, post‐tonsillectomy hemorrhage, tonsillectomy

## Abstract

**Objectives:**

Post‐tonsillectomy hemorrhage (PTH) is a common complication in pediatric patients, yet management strategies vary due to limited outcome data. Historically, all patients at our institution with postoperative bleeding or visible clot were taken to the OR. In 2018, a new protocol was introduced in which patients with a visible clot but no active bleeding were managed conservatively. This study evaluates the efficacy and revisit outcomes of this observational approach versus immediate surgical intervention.

**Methods:**

A retrospective cohort study was conducted at a tertiary center over two 3‐year periods. Pediatric patients (< 18 years) presenting to the ED (emergency department) with PTH were categorized into pre‐protocol (*n* = 86, surgical) and post‐protocol (*n* = 134, observational) cohorts. Outcomes included revisit rates, surgical intervention rates, and hospital length of stay. Statistical analysis was performed using two‐sample *t* tests and Fisher's exact tests.

**Results:**

The post‐protocol cohort had a higher proportion of patients presenting with PTH (3.86% vs. 5.88%, *p* = 0.0018), but fewer underwent surgery (54.65% vs. 26.87%, *p* < 0.0001). No significant differences were found in repeat visits leading to surgery or multiple surgical interventions. More patients with a visible clot avoided surgery post‐protocol (3.49% vs. 29.85%, *p* < 0.0001). However, the average hospital stay was longer (9.25 vs. 13.13 h, *p* = 0.0774).

**Conclusion:**

An observational approach for pediatric PTH patients with clot‐only presentation significantly reduced the need for surgical intervention. This strategy may minimize unnecessary procedures and improve care quality.

**Level of Evidence:**

3.

## Graphical abstract

This study compares outcomes of observational versus surgical management of pediatric post‐tonsillectomy hemorrhage. We found that observational management was safe and avoided unnecessary procedures in selected cases. These results support tailoring intervention to exam findings to optimize patient care.

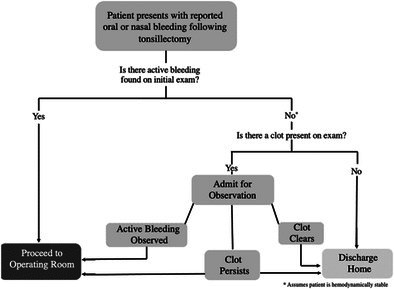

## 1 Introduction

Tonsillectomy is one of the most commonly performed operations on pediatric patients in the United States, with over 500,000 cases performed each year on patients under 15 years of age [1]. While it is a routine and effective procedure, it is associated with significant postoperative complications such as pain, dehydration, and hemorrhage [2]. Of these, post‐tonsillectomy hemorrhage (PTH) is one of the most common complications, occurring with an incidence of 2%–7.4% [3, 4, 5]. It is relatively common for patients to present to the emergency department (ED) following tonsillectomy, reporting symptoms of oral bleeding [6]. PTH is classified into primary or secondary PTH, based on whether the bleeding occurs < 24 h after surgery and is a result of immediate postoperative hemorrhage, or if it occurs > 24 h after surgery and is due to the dislodgment of the eschar. Secondary PTH commonly occurs 5–12 days following tonsillectomy [4, 7].

PTH is a serious life‐threatening complication due to the potential for airway obstruction, aspiration, hypovolemic shock, and death [7, 8]. Even with the potential severity of the complications, management of patient bleeding is debated among otolaryngologists, and universal guidelines do not currently exist. Surveys of otolaryngologists have shown a lack of consensus with regard to managing patients with a history of bleeding but a normal exam or patients who present with a clot within the tonsillar fossa (but not actively bleeding) [2, 9]. The lack of guidelines poses risks to patient safety due to wide variation in management strategies, and understanding appropriate ways to avoid this risk is essential to provide the best care to patients [10]. Additionally, avoiding operative intervention has implications for patient and hospital expenditures [11].

Much of the historical data surrounding PTH has centered around surgical technique, predictive patient factors, and postoperative pain control regimen as predisposing factors for bleeding [8, 12, 13, 14, 15]. Patient outcomes analysis based on these metrics has been well documented and can be helpful when determining whether to operate on or observe a patient [12, 13, 14, 15, 16, 17].

Though we understand predictive factors for PTH, there is a paucity of data on how various management strategies for patients presenting with PTH affect patient outcomes and re‐bleed rates. It has been noted by numerous authors that establishing best practice would be helpful in building universal practice recommendations [2, 6, 9, 17]. Therefore, it is necessary to evaluate current PTH treatment strategies—specifically, understanding the risks and benefits of observation versus surgical management—to better understand the impact on patient outcomes.

Algorithms for managing patients with PTH generally stratify patients based on history of the bleeding episode, physical exam at presentation, and hemodynamic status. For many years at our institution, the management approach was more active or aggressive, with immediate surgical intervention for all pediatric patients with PTH with active bleeding or a clot present (but not actively bleeding). Those without bleeding or clot were observed for a short period and then discharged home.

**FIGURE 1:**
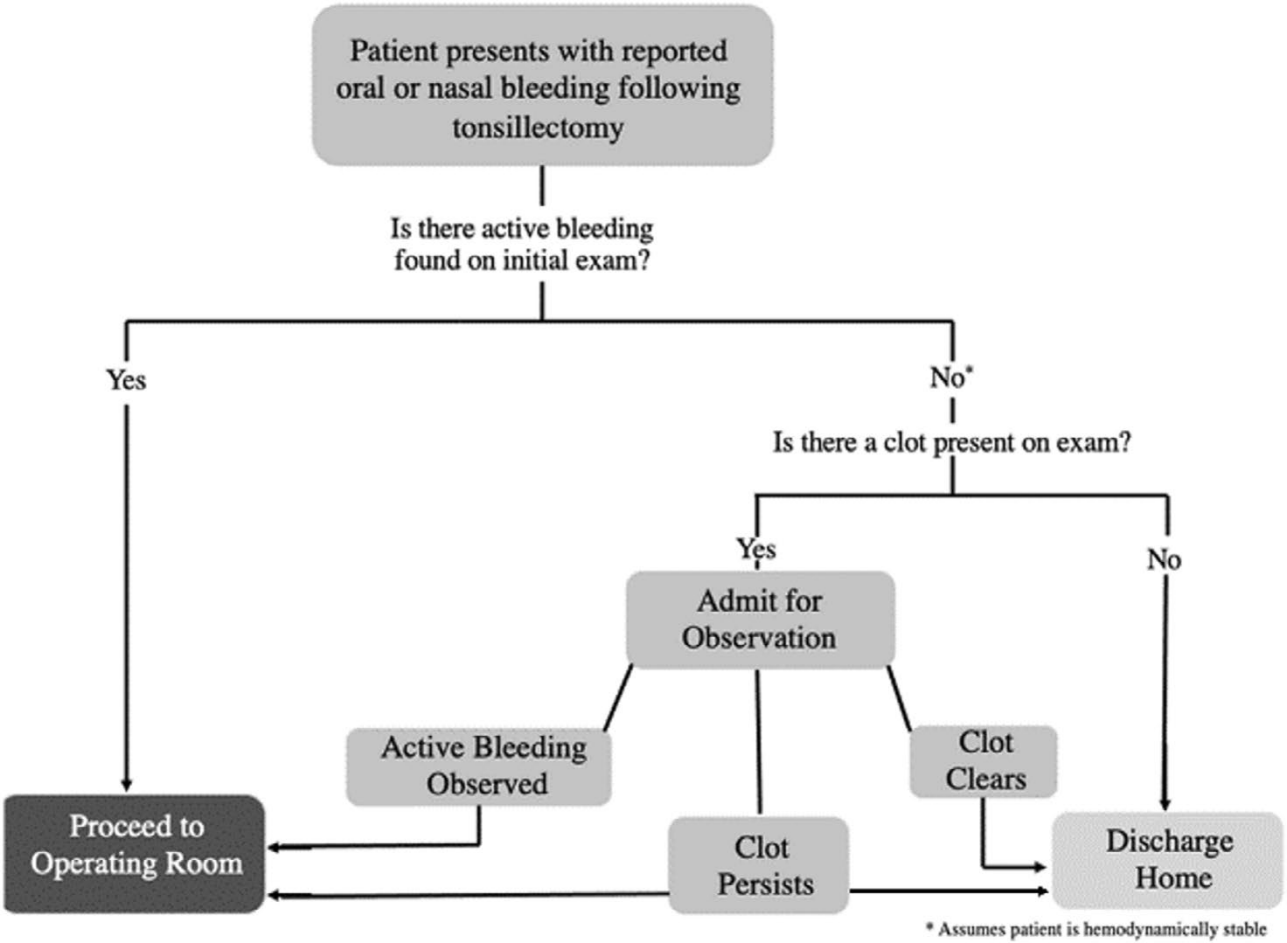
Post‐protocol treatment algorithm.

Several years ago, a more conservative observational management strategy was implemented in the hopes of avoiding operative intervention for a subset of patients presenting with PTH—specifically, patients with a clot present in the oropharynx (but without active bleeding). The more detailed algorithm (Figure 1) included evaluation of the patient by an otolaryngology resident, fellow, or attending physician; the patient is then stratified by the history, physical exam findings, and hemodynamic status. All patients with active bleeding visualized on exam or with hemodynamic instability are transported to the operating room (OR) for emergent surgical intervention. Patients with a history of bleeding prior to presentation, but no active bleeding, normal appearing post operative eschar, and no visualized clot in the tonsillar fossae are observed in the ED for 1–3 h and possibly admitted depending on patient history and lab values. Provided no bleeding during the observation period, these patients are then discharged home. Patients who have no active bleeding on exam, but have a clot present in the oropharynx, are usually observed overnight on the inpatient otolaryngology service on a clear liquid diet. On follow up examination 6–8 h post‐presentation, if the clot has cleared, they are discharged home. If they have significant retained clot and possibility for rebleed is suspected, they are transported non‐emergently to the OR for exam under anesthesia, clot removal, and control of oropharyngeal hemorrhage. Management of patients presenting with recurrent episodes of bleeding (or other atypical situations) is left to the clinical judgment of the evaluating physician.

The goal of this study is to evaluate this proposed algorithm for the management of pediatric PTH to better understand the true need for OR utilization for pediatric patients presenting with PTH. Many institutions have analyzed their management protocols; yet no one has been able to compare their outcomes to a control. This study is among the first to use a historical cohort as the control to better compare outcomes. We hypothesized that this algorithm, utilizing close observation rather than initial operative intervention, would minimize the need for operative intervention in benign cases and therefore minimize healthcare utilization and decrease anesthetic risk.

## 2 Materials and Methods

### 2.1 Patient Selection

Patients 18 years or younger who underwent tonsillectomy with an otolaryngologist at a tertiary academic medical center between 1/1/2013 and 7/1/2016, and between 1/1/2019 and 7/1/2022 were included in this study. The conservative observational protocol was first utilized in fall of 2016, but full adoption by all faculty members was not completed until approximately summer of 2018, thus the 7/1/2016 to 1/1/2019 washout period. We used the data from 1/1/2013 to 7/1/2016 as a historical control period and the data from 1/1/2019 to 7/1/2022 as the experimental group, which we named pre‐protocol and post‐protocol groups, respectively. Cases were identified within the electronic medical record (Epic Clarity; Epic Systems Corporation). Surgeries were identified by *Current Procedural Terminology* (CPT) codes associated with tonsillectomies performed by an otolaryngologist. All tonsillectomies were performed with an extracapsular technique. These codes included 42,820 (adenotonsillectomy under the age of 12), 42,821 (adenotonsillectomy over the age of 12), 42,825 (tonsillectomy under the age of 12), and 42,826 (tonsillectomy over the age of 12). All ED encounters occurring between > 24 h and 15 days of surgery were included for analysis of postoperative complications. Fifteen days was chosen as the cutoff to capture the most likely time for secondary tonsil hemorrhage as well as any outliers. These encounters were then sorted by the type of complication. Patients with multiple encounters were analyzed by their first ED visit, unless they returned to the OR at a subsequent visit in which that encounter was used for encounter specific‐data (such as hematologic labs). For this study, PTH was defined as any reports of bleeding that occurred following tonsillectomy, excluding epistaxis, whether reported at home from family, reported by a medical provider, or witnessed by an ED or otolaryngology physician. Notably, most of the patients had reports of bleeding per family, but no active bleeding on ED evaluation. Exclusion criteria included age above 18, ED visit not related to bleeding, ED visits outside of our institution's ED, inaccurate or incomplete documentation, and epistaxis. Patient demographics, perioperative variables, and postoperative outcomes were collected by manual chart review. The study was approved by the University of Wisconsin Health Sciences IRB.

### 2.2 Outcomes of Interest

The primary outcomes of interest were overall percent of patients requiring surgical intervention, percent of patients with multiple returns to the OR, percent of patients with multiple ED visits, and percent of patients presenting with a clot that needed operative intervention. Secondary outcomes included PTH rates and patient length of stay. Demographics, clinical management, and patient outcomes were compared for all patients in the study between the two cohorts.

### 2.3 Statistical Analysis

The demographic data was analyzed using descriptive statistics including frequencies, mean, and standard deviation (SD). A two‐sample *t* test was used to determine the significance of continuous variables including age, lab values, and lengths of time. Two‐tailed *t* test statistical analysis and data visualizations were performed using Microsoft Excel for Microsoft 365 MSO (Version 2307 Build 16.0.16626.20086). Fisher's exact test was utilized to determine the significance of differences between patient percentages between the two cohorts. The significance level was set at a *p* value (*p*) less than or equal to 0.05. Fisher's exact test was performed using the GraphPad QuickCalcs website: https://www.graphpad.com/quickcalcs/contingency1/ (accessed August 2023).

## 3 Results

During the period of 1/1/2013 to 7/1/2016 (pre‐protocol cohort), 2229 tonsillectomies were performed. In this timeframe, 161 post‐tonsillectomy ED encounters were identified in patients < 18 years old. Fifty‐eight encounters were excluded for being unrelated to PTH. Three encounters were excluded for being < 24 h post‐surgery. Two encounters were excluded for inadequate documentation. After exclusions, there remained 98 encounters occurring in 86 patients (10 encounters were singular additional visits for 10 patients and 2 were recurrent ED visits for one patient).

During the period of 1/1/2019 to 7/1/2022 (post‐protocol cohort), 2277 tonsillectomies were performed. In this timeframe, 286 ED encounters were identified in patients < 18 years old. A total of 125 were excluded for epistaxis or not being ED encounters for symptoms of bleeding, and 12 encounters were excluded for ED presentations outside of the study institution, incomplete exam, ED encounter < 24 h post‐tonsillectomy, or poor documentation. After exclusions, there remained 149 encounters occurring in 134 patients (7 patients had a single additional visit to the ED, 1 patient had two additional visits, and 2 patients had 3 additional visits).

Table 1 shows the patient characteristics of the pre‐ and post‐protocol cohorts. The number of patients presenting with bleeding differed significantly between the pre‐protocol and post‐protocol cohorts (pre: *n* = 86 vs. post: *n* = 134, *p* = 0.0018). However, there was no statistically significant difference in the average age of patients between the two cohorts (pre: 7.53 vs. post: 7.40, *p* = 0.8207). The post‐protocol cohort was composed of 76 (56.72%) females and 58 (43.28%) males. In terms of race, a statistically significant difference was observed in the distribution of patients who identified as white (pre: 94.19% vs. post: 85.07%, *p* = 0.0491). Indication for surgery demonstrated significant differences in the proportions of patients with chronic/recurrent tonsillitis (pre: 25.58% vs. post: 8.96%, *p* = 0.0011) and patients undergoing surgery for sleep‐disordered breathing/obstructive sleep apnea (SDB/OSA) (pre: 62.79% vs. post: 76.12%, *p* = 0.0475). The distribution of surgical technique in PTH patients showed significant changes between the pre‐ and post‐protocol cohorts, with notable increases in the frequency of hot (dissection & tonsil removal entirely with monopolar and bipolar cautery) tonsillectomy (pre: 2.33% vs. post: 47.01%, *p* = 0.0001) and decreases in cold (dissection & tonsil removal with cold instruments followed by bipolar cautery for hemostasis) tonsillectomy (pre: 50.00% vs. post: 23.88%, *p* = 0.0001). Bipolar and coblation techniques also showed variations, but the differences were not statistically significant. The overall prevalence of surgical technique, indication, and medication use for all tonsillectomies performed during each period was not assessed in this study. Hematologic lab values indicated no statistically significant differences in hematocrit, hemoglobin, platelet count, activated partial thromboplastin time (aPTT), and international normalized ratio (INR) between the Pre‐protocol and post‐protocol cohorts. Postoperative pain medication use differed between cohorts (Table 1). Ibuprofen use increased significantly post‐protocol (74.42% vs. 97.01%, *p* < 0.0001), while acetaminophen use remained unchanged (100.00% vs. 99.25%). Methylprednisolone (8.21%) and naproxen (0.75%) were only rarely used in the post‐protocol cohort. Oxycodone use increased in the post‐protocol cohort non‐significantly (84.88% vs. 91.79%, *p* = 0.1240).

**TABLE 1:** Comparison of patient characteristics.

Patient characteristics			
	Pre‐protocol	Post‐protocol	*p*
Number of patients presenting with bleeding, *n* [% of total tonsillectomies performed]	86 [3.86]	134 [5.88]	0.0018
Average age [SD]	7.53 [4.10]	7.40 [4.54]	0.8207
Race, *n* [%]			
White	81 [94.19]	114 [85.07]	0.0491
Black or African American	4 [4.65]	12 [9.00]	0.2931
American Indian or Alaska Native	0 [0.00]	2 [1.50]	
Asian	1 [1.16]	4 [3.00]	0.6507
Patient declined to answer	0 [0.00]	2 [1.50]	
Gender, *n* [%]			
Female	46 [53.49]	76 [56.72]	0.6778
Male	40 [46.51]	58 [43.28]	
Indication for surgery, *n* [%]			
SDB/OSA	54 [62.79]	102 [76.12]	0.0475
Chronic/recurrent tonsillitis	22 [25.58]	12 [8.96]	0.0011
SDB/OSA + chronic/recurrent tonsillitis	10 [11.63]	16 [11.94]	1.0000
SDB/OSA + other	0 [0.00]	2 [1.49]	
Other	0 [0.00]	2 [1.49]	
Surgical technique, *n* [%]			
Hot	2 [2.33]	63 [47.01]	0.0001
Cold	43 [50.00]	32 [23.88]	0.0001
Bipolar	32 [37.21]	33 [24.63]	0.0504
Coblation	9 [10.47]	6 [4.48]	0.1032
Hematologic lab values, *n* [average, SD]			
Hematocrit	17 [30.13, 1.43]	42 [33.16, 1.20]	0.1547
Hemoglobin	18 [12.52, 1.48]	45 [15.97, 18.04]	0.4335
Platelet count	18 [339.33, 29.56]	42 [370.86, 115.26]	0.3482
aPTT	10 [31.05, 3.36]	21 [33.73, 5.94]	0.1960
INR	12 [1.075, 0.06]	21 [1.1, 0.02]	0.4747
Medications, *n* [%]			
Ibuprofen	64 [74.42]	130 [97.01]	< 0.0001
Acetaminophen	86 [100]	133 [99.25]	
Methylprednisolone	0 [0]	1 [0.75]	
Naproxen	0 [0]	1 [0.75]	
Oxycodone	73 [84.88]	123 [91.79]	0.5022

Abbreviations: OSA, obstructive sleep apnea; SD, standard deviation; SDB, sleep disordered breathing.

Table 2 shows the average time until bleeding occurrence and length of stay for PTH patients. The average time to bleeding presentation from initial tonsillectomy to the first ED encounter was similar between the cohorts (pre: 6.02 days vs. post: 5.80 days, *p* = 0.5622). This is consistent with previous data depicting an average time to ED presentation for PTH to be approximately 6 days [5]. The average total length of stay for patients with a clot and no active bleeding was longer in the Post‐protocol cohort (pre: 9.25 h vs. post: 13.13 h, *p* = 0.0774).

**TABLE 2:** Comparison of patient length of stay and time to first emergency room visit.

	Pre‐protocol	Post‐protocol	*p*	Mean difference	95% Confidence interval
Average time from surgery to first ED encounter, days [SD]	6.02 [3.03]	5.80 [2.64]	0.5622	−0.22	[−1.01, 0.57]
Average total length of stay for non‐OR patients, hours [SD]	6.11 [7.51]	9.25 [7.92]	0.0353	3.14	[1.03, 5.25]
Average total length of stay for clot patients, hours [SD]	9.25 [6.47]	13.13 [11.16]	0.0774	3.88	[0.00, 7.76]

Abbreviations: ED, emergency department; SD, standard deviation.

Table 3 displays patient outcomes. The post‐protocol cohort exhibited a higher percentage of patients presenting to the ED with bleeding symptoms compared to the pre‐protocol cohort (pre: 3.86% vs. post: 5.88% *p* = 0.0018). The post‐protocol cohort showed a reduction in the proportion of patients requiring surgical intervention for PTH. Specifically, 36/134 patients (26.87%) in the post‐protocol cohort underwent surgery compared to 47/86 patients (54.65%) in the pre‐protocol cohort (*p* < 0.0001, odds ratio = 0.30, 95% confidence interval: 0.17, 0.54). There were no significant differences between the cohorts in terms of patients requiring repeat surgical interventions or patients requiring surgical intervention after a previous non‐operative ED encounter. Of patients presenting with bleeding symptoms, active bleeding rates were similar between the cohorts (pre: 15.12% vs. post: 14.93%, *p* = 1.0000).

**TABLE 3:** Comparison of patient outcomes.

	Pre‐protocol				Post‐protocol						
Patient outcomes	Number of patients	Percent of total tonsillectomies	Percent of patients who presented with bleeding symptoms	Percent of total patients presenting with a clot at any visit	Number of patients	Percent of total tonsillectomies	Percent of patients who presented with bleeding symptoms	Percent of total patients presenting with a clot at any visit	*p*	Odds ratio	95% Confidence interval
Total tonsillectomies performed	2229				2277						
Total patients presenting with bleeding symptoms	86	3.86%			134	5.88%			*p* = 0.0018	1.56	(1.18, 2.06)
Went to OR post‐tonsillectomy	47	2.11%	54.65%		36	1.58%	26.87%		*p* < 0.0001	0.30	(0.17, 0.54)
Active Bleed	13	0.58%	15.12%		20	0.88%	14.93%		*p* = 1	0.99	(0.46, 2.10)
Went to OR and Returned to ED	5	0.22%	5.81%		1	0.04%	0.75%		*p* = 0.0349	0.12	(0.01, 1.06)
Went to OR on a recurrent visit	7	0.31%	8.14%		5	0.22%	3.73%		*p* = 0.2235	0.43	(0.13, 1.42)
Went to OR twice total	4	0.18%	4.65%		1	0.04%	0.75%		*p* = 0.0778	0.15	(0.02, 1.40)
Went to OR once after a previous ED visit	3	0.13%	3.49%		4	0.18%	2.99%		*p* = 1.0000	0.85	(0.19, 3.90)
Had > 1 ED visit for bleed symptoms	11	0.49%	12.79%		10	0.44%	7.46%		*p* = 0.2403	0.55	(0.22, 1.36)
Patients who presented with a clot	32	1.44%	37.21%		54	2.37%	40.30%		*p* = 0.6732	1.14	(0.65, 1.99)
Ultimately required OR	29	1.30%	33.72%	90.63%	14	0.61%	10.45%	25.93%	*p* = 0.0001	0.0362	(0.01, 0.14)
Had > 1 OR Return	3	0.13%	3.49%	9.38%	1	0.04%	0.75%	1.85%	*p* = 0.1431	0.18	(0.02, 1.83)
Did not go to OR	3	0.13%	3.49%	9.38%	40	1.76%	29.85%	74.07%	*p* < 0.0001	27.62	(7.26, 105.00)
Had a return visit to ED	2	0.09%	2.33%	6.25%	2	0.09%	1.49%	3.70%	*p* = 0.6262	0.58	(0.08, 4.31)
Bedside suction/cautery used	1	0.04%	1.16%	3.13%	0	0.0%	0.0%	0.0%	*p* = 0.3721		

Abbreviations: ED, emergency department; OR, operating room.

Although a larger proportion of patients who presented with bleeding symptoms had a clot on exam in the Post‐protocol cohort (54 patients, 40.30%) compared to the Pre‐protocol cohort (32 patients, 37.21%), this difference was not statistically significant (*p* = 0.6732) (Table 3). However, significantly fewer patients who presented with a clot to the ED in the Post‐protocol cohort ultimately underwent operative intervention (14/54 patients, 25.93%) compared to the pre‐protocol cohort (29/32 patients, 90.63%) (*p* = 0.0001, odds ratio = 0.0362, 95% confidence interval: 0.01, 0.14). The pre‐protocol cohort did have a higher percentage of patients with multiple OR visits post‐tonsillectomy (pre: 4.65% vs. post 0.75%); however, this was not statistically significant (*p* = 0.0778). The usage of bedside suction/cautery in the ED was negligible in both cohorts, with only one patient (0.75%) in the Pre‐protocol cohort and none in the Post‐protocol cohort undergoing this intervention (*p* = 0.3721). Of the 29 pre‐protocol patients who had a clot and went to the OR, 3 came back to the ED with bleeding symptoms, and of the 40 post‐protocol patients who had a clot present and did not go to the OR, only 2 had an ED revisit (pre: 10.34% vs. post: 5.0%, *p* = 0.6431).

## 4 Discussion

PTH is a common complication following tonsillectomy and can lead to significant morbidity and healthcare utilization. This study investigated the outcomes of a conservative management approach compared to prompt surgical intervention. The primary focus was management of patients presenting with a clot in the tonsillar fossa on ED physical exam but with no active bleeding. Prior studies have attempted to describe the outcomes of a conservative management protocol; however, improvement in patient outcomes by comparing to a retrospective control cohort has not previously been described [5, 6, 12, 14].

In this study, the average times from surgery to the day of first ED presentation, frequency of PTH, and percent of patients presenting with bleeding symptoms that were actively bleeding were similar to ranges described in previous studies [5, 13, 16, 18]. Varying rates are described within the literature, and a likely rationale, which has been proposed by previous studies, could be the lack of consistent classification or definitions of PTH [5, 19]. The difference in overall percent of patients presenting with bleeding symptoms between the two cohorts is possibly due to the differences in operative technique, primary indication for tonsillectomy, or in pain control regimens. This is speculative as we do not have data from every tonsillectomy performed to assess overall frequency. However, we did notice differences between the two cohorts in these factors. This points to the possibility of differences in baseline characteristics of the larger sample of all patients receiving tonsillectomy during this period that may impact bleeding between the two cohorts. It may also be related to increased parental education on signs to watch for or possibly a lower threshold for coming to the ED for evaluation. It is reasonable that the COVID‐19 pandemic played a role in changes in the likelihood of patients presenting for concerns during the post‐protocol period due to heightened health anxiety and awareness. Though any of these factors could reasonably explain the differences in bleeding rates between the cohorts, our bleeding rates are similar to a large cohort study evaluating ED bleed visit rates, finding the 50th and 95th percentiles for post‐tonsillectomy bleeding were 1.97% and 4.75% [8]. The most common indication for tonsillectomy in our cohorts was OSA/SDB, which is consistent with previous studies [5, 6, 12, 20].

Much of the current literature describing PTH protocols has focused on predictive factors like surgical technique, indication, medication use, and age for PTH [6, 12, 13, 14, 16, 17, 18]. Awareness of who is at risk of PTH is essential; however, the question remains of how we manage the patients who do ultimately present to the ED with symptoms of PTH, specifically those patients presenting with a clot without active bleeding on examination. As more institutions transition to conservative protocols, understanding the impact on patient outcomes is necessary.

For patients presenting with a clot (but not actively bleeding), the rates of recurrent bleeding and return ED visits were not significantly different between the Pre‐ and Post‐protocol cohorts in our study. This is in agreement with previous retrospective studies comparing management with observation versus surgical intervention [5, 21]. Thus, for patients presenting with a clot, a conservative observational approach may be optimal as the risks and costs of additional surgery and anesthesia exposure may frequently be avoided.

These findings indicate that the conservative observational approach led to a substantial reduction in the proportion of patients requiring surgical intervention in cases of PTH. This reduction was primarily related to a significant change in management in a specific subset of patients—those presenting with a clot but without active bleeding. These findings highlight the benefits of the conservative management approach that have also been described in previous studies [5, 6, 14, 17]. Our data suggest that a conservative management strategy effectively identifies patients who can be managed with observation. This is consistent with the trend in conservative management that other institutions have described [2, 5, 12, 14, 17].

The strengths of this study include being among the first retrospective cohort studies for depicting patient outcomes who were managed by different PTH protocols. However, certain limitations should be acknowledged, including uncertain prevalences in surgical technique and indication, the presence of a pandemic during the post‐protocol period, as well as non‐standardized pain medications prescribed for all patients who underwent tonsillectomy in the pre‐ and post‐protocol cohorts. This is a potential source of bias due to a heterogeneity in baseline patient characteristics between the pre‐ and post‐protocol cohorts. There are also differences in patient characteristics for those specifically presenting with bleeding symptoms. These differences in surgical technique, indication, and pain regimen may have also impacted re‐bleed rates or the need for operative intervention. The study design was retrospective, introducing potential biases and limitations in data collection. Furthermore, the study was conducted at a single institution, which may limit the generalizability of the findings to broader populations. Finally, the adoption of the post‐protocol management strategy was gradual; however, the 3‐year washout period between cohorts attempted to minimize possible crossover between cohorts.

## 5 Conclusion

In conclusion, this study provides valuable insights into the effectiveness of a conservative management approach for PTH, particularly in patients presenting with a clot on physical exam. This patient management protocol was associated with a significant reduction in the need for surgical interventions, suggesting that it offers a superior alternative to active surgical management for this patient population. Future prospective studies involving multiple centers would be needed to validate these findings and provide a more comprehensive understanding of the benefits and limitations of this approach in diverse patient populations. In addition, a comprehensive meta‐analysis of current patient management strategies and their patient outcomes is essential to provide a framework to guide institutions to improve patient safety and decrease healthcare costs.

## Conflicts of Interest

The authors declare no conflicts of interest.

## Data Availability

The data that support the findings of this study are available from the corresponding author upon reasonable request.
